# Sex Differences in Neurovascular Control: Implications for Obstructive Sleep Apnea

**DOI:** 10.3390/ijms241713094

**Published:** 2023-08-23

**Authors:** Joshua M. Bock, Ian M. Greenlund, Virend K. Somers, Sarah E. Baker

**Affiliations:** 1 Department of Cardiovascular Medicine, Mayo Clinic, Rochester, MN 55901, USA; bock.joshua@mayo.edu (J.M.B.);; 2 Department of Anesthesiology and Perioperative Medicine, Mayo Clinic, Rochester, MN 55901, USA

**Keywords:** sympathetic, neural, blood pressure, sex, adrenergic

## Abstract

Patients with obstructive sleep apnea (OSA) have a heightened risk of developing cardiovascular diseases, namely hypertension. While seminal evidence indicates a causal role for sympathetic nerve activity in the hypertensive phenotype commonly observed in patients with OSA, no studies have investigated potential sex differences in the sympathetic regulation of blood pressure in this population. Supporting this exploration are large-scale observational data, as well as controlled interventional studies in healthy adults, indicating that sleep disruption increases blood pressure to a greater extent in females relative to males. Furthermore, females with severe OSA demonstrate a more pronounced hypoxic burden (i.e., disease severity) during rapid eye movement sleep when sympathetic nerve activity is greatest. These findings would suggest that females are at greater risk for the hemodynamic consequences of OSA and related sleep disruption. Accordingly, the purpose of this review is three-fold: (1) to review the literature linking sympathetic nerve activity to hypertension in OSA, (2) to highlight recent experimental data supporting the hypothesis of sex differences in the regulation of sympathetic nerve activity in OSA, and (3) to discuss the potential sex differences in peripheral adrenergic signaling that may contribute to, or offset, cardiovascular risk in patients with OSA.

## 1. Introduction

In 2022, the American Heart Association released an advisory document detailing eight domains of everyday life, titled Life’s Essential 8, which should be optimized to reduce cardiovascular risk [1]. This report built upon the prior iteration, Life’s Simple 7 [2], to include sleep as a key regulator of cardiovascular health. In this light, blood pressure is among the most widely studied markers of cardiovascular risk and is modulated by sleep [3]. That is, both habitual short sleep duration and experimental sleep restriction are associated with increased blood pressure in otherwise healthy individuals [3]. Of the various mechanisms that modulate blood pressure, the sympathetic nervous system is particularly influential in the context of sleep-related changes. Experimental sleep restriction [4] and studies using total sleep deprivation [5] report robust activation of the sympathetic nervous system. Interestingly, studies at the population level and smaller controlled trials indicate the detrimental effects of short sleep, as well as intermittent sleep disruption, are more pronounced in females relative to males [6,7].

In addition to healthy adults, increased sympathetic nerve activity is also reported in many sleep pathologies [8], with the most studied sleep condition being obstructive sleep apnea (OSA). Characterized by intermittent reductions in airflow with resulting hypercapnic hypoxemia during sleep, OSA is associated with both the development of hypertension as well as tonic elevation of sympathetic nerve activity [9]. However, OSA is also considered male-dominant [10], and, to date, no study has examined potential sex differences in the regulation of sympathetic nerve activity in this population. Thus, the purpose of this article is to review the literature on sex differences in cardiovascular risk attributable to poor sleep, sympathetic reflex regulation of arterial blood pressure, and peripheral regulatory mechanisms of sympathetic transduction to the vasculature. We hope this article will serve as an impetus for the exploration of sex differences in sleep-induced changes in neurovascular physiology.

## 2. Sex Differences in Neurovascular Control of Blood Pressure

Young females are protected against developing hypertension and other cardiovascular diseases relative to young males. With aging, this risk increases in both sexes; however, the rate of rise in cardiovascular risk is greater in females than males. Specifically, by the approximate age of menopause, the incidence of hypertension becomes similar to, or exceeds, that of males of the same age [11,12]. There are fundamental sex differences in blood pressure regulation that underly these observations. Young males control blood pressure through a reciprocal relationship between cardiac output and sympathetic control of total peripheral resistance. For instance, males with high resting sympathetic activity will have low cardiac output and vice versa to maintain blood pressure [13]. As this reciprocal relationship is not seen in young females [13], it is likely that sex differences exist in peripheral adrenergic signaling.

### 2.1. α-Adrenergic Signaling 

**Figure 1:**
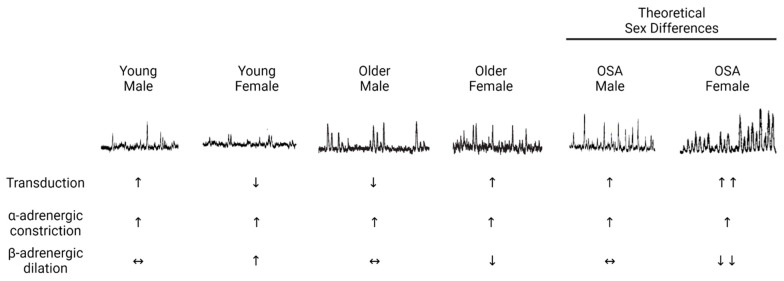
Neurograms of sympathetic nerve activity recorded via microneurography under resting conditions in humans. Sympathetic nerve activity tends to be lower in young females relative to young males [13]; however, with aging, this pattern is reversed [19,20]. While patients with obstructive sleep apnea (OSA) have increased sympathetic nerve activity [21], there have been no studies examining potential sex differences in humans. Based upon studies indicating that females experience more deleterious effects on sympathetic control of blood pressure following sleep disruption [5,22], and the pattern of oxygen desaturation in females with OSA [23], we hypothesize that females with OSA have greater sympathetic nerve activity than males with OSA (theoretical sex differences). Transduction summarizes the vasoconstrictive response to a given burst of sympathetic nerve activity; upward arrows indicate more vasoconstriction, whereas downward arrows are indicative of less vasoconstriction. Young females have less neurovascular transduction as compared to young males, although this dissipates with aging, whereby older females have a more pronounced vasoconstrictive response to sympathetic nerve activity relative to older males [18]. As blood pressure elevations in OSA are attributable to increased sympathetic nerve activity [24], and we hypothesize that sympathetic nerve activity is greater in females with OSA, we anticipate that neurovascular transduction is increased in females with OSA relative to males with OSA. α-adrenergic constriction and β-adrenergic dilation refer to changes in arterial diameter in response to neurotransmitters, such as norepinephrine, binding to these adrenergic receptors. Young females demonstrate less vasoconstriction in response to intra-arterial infusion of α-adrenergic-specific pharmacological agents (e.g., phenylephrine, clonidine) relative to males [15]. Concomitantly, greater β-adrenergic-mediated vasodilation is observed in young females relative to young males [18]. However, with advancing age, β-adrenergic-mediated dilation is less pronounced in females but not males [25,26]. To date, no study has directly measured arterial diameter during intra-arterial drug infusions in patients with OSA. Based on the literature, we anticipate that α-adrenergic-mediated vasoconstriction is similar between females and males with OSA, but that β-adrenergic-mediated vasodilation is selectively attenuated in females with OSA.

Several lines of evidence demonstrate that young females have blunted peripheral α-adrenergic-mediated vasoconstriction relative to young males (Figure 1). First, young females have less vasoconstriction than young males in response to intra-arterial infusions of the neurotransmitter norepinephrine [14]. In response to more selective alpha-adrenergic agonists, specifically phenylephrine (primarily α_1_) and clonidine (primarily α_2_), males demonstrated vasoconstrictor responses, whereas females did not have significant vasoconstriction [15]. Second, studies using pharmacological blockade of the autonomic ganglia with trimethaphan demonstrate smaller decreases in the blood pressure of young females relative to young males [16]. This is indicative that young females have less autonomic support of blood pressure than young males. In males, an inverse relationship between sympathetic activity and the change in blood pressure during ganglionic blockade was observed, whereby males with the greatest sympathetic activity also had the largest reductions in blood pressure [17]. Interestingly, this relationship between changes in sympathetic nerve activity and blood pressure during ganglionic blockade was not present in females [16], alluding to the presence of a buffer between sympathetic activity and peripheral vasoconstriction. This buffer was confirmed in subsequent work describing sex differences in neurovascular transduction (i.e., the magnitude of change in blood pressure for a given burst of sympathetic activity), where young females had less neurovascular transduction relative to young males [18] (Figure 1). The root cause of the sex differences in neurovascular transduction remains unclear but almost certainly involves α-adrenergic signaling (e.g., neurotransmitter release or reuptake; receptor density or sensitivity). In addition to the sex differences in peripheral α-adrenergic signaling described above, sex differences also exist in β-adrenergic signaling.

### 2.2. β-Adrenergic Signaling

Multiple studies have demonstrated that augmented β-adrenergic signaling plays an important role in regulating arterial blood pressure in young females. As mentioned above, there is no relationship between sympathetic nerve activity and total peripheral resistance in young females. However, a significant relationship emerges under β-adrenergic receptor blockade via propranolol such that females have a similar relationship between these variables as males [13]. These data suggest that β-adrenergic receptor-mediated vasodilation offsets the relationship between sympathetic activity and total peripheral resistance in young females but not in males (Figure 1). In addition, young females have very low vasoconstrictor responses during intra-arterial infusions of norepinephrine [14]. However, the vasoconstrictor responses to norepinephrine in young females are significantly greater during β-adrenergic receptor antagonism with propranolol, such that young females have similar vasoconstrictor responses as young males [14]. Furthermore, β-adrenergic blockade in young females results in a significant increase in the vascular response to sympathetic nerve activity (neurovascular transduction) [18]. Finally, there are direct data reporting augmented vasodilation during intra-arterial infusion of β-adrenergic agonists in young females relative to young males [27]. Together, these studies highlight the importance of β-adrenergic receptor-mediated vasodilation in regulating the blood pressure of young females by offsetting α-adrenergic receptor-mediated vasoconstriction in the peripheral vasculature. Practical applications of the relationships between peripheral adrenergic receptors in young females include the heightened fainting prevalence in young females and lower blood pressure in young females compared to age-matched young males; importantly, these effects persist throughout life and continue through menopause [28].

### 2.3. Changes with Aging

Several changes in blood pressure regulation contribute to the age-associated rise in blood pressure, hypertension, and cardiovascular risk. Direct measurements of sympathetic nerve activity demonstrate increases with age in both males and females [19,20] (Figure 1). Further, there is an age-related increase in the autonomic support of blood pressure that is driven by increased sympathetic and decreased parasympathetic nerve activity [29,30,31]. This contributes to the “double hit” to blood pressure regulation in females around the age of menopause, where there is an accelerated increase in blood pressure and risk of hypertension. This double hit is characterized by (1) the increase in sympathetic nerve activity and (2) a reduction in β-adrenergic receptor-mediated vasodilation. During infusion of non-selective β-adrenergic agonists, as well as β_2_-adrenergic-specific agonists, postmenopausal females demonstrate blunted vasodilatory responses relative to young, premenopausal females [25,26]. Unlike young females, postmenopausal females exhibit significant vasoconstrictor responses to norepinephrine, which is not altered by co-infusion with the β-adrenergic antagonist propranolol [14]. These data suggest that β-adrenergic receptor-mediated vasodilation no longer offsets α-adrenergic receptor-mediated vasoconstriction in postmenopausal females. Finally, there is also evidence that the blood pressure response to a given level of sympathetic nerve activity (neurovascular transduction) changes with aging [18]. In males, neurovascular transduction decreases with age such that age-associated increases in sympathetic nerve activity are buffered, as evidenced by a lower vasoconstrictor response. Conversely, in females, neurovascular transduction increases with age, manifesting as an exaggerated vascular response to sympathetic activity. Further, this increase in neurovascular transduction is not improved with β-adrenergic blockade with propranolol, suggesting that β-adrenergic-mediated vasodilation no longer offsets α-adrenergic-mediated vasoconstriction in postmenopausal females [18]. 

In addition to the increased sympathetic activity and changes in peripheral signalling that are associated with aging, changes in the firing patterns of sympathetic neurons have also been described. Historically, muscle sympathetic nerve activity has been quantified in vivo as multi-unit bursts of an integrated signal. Recent work from Shoemaker and colleagues has allowed the study of post-ganglionic sympathetic nerve firing in humans. This is methodologically possible by recording sympathetic nerve activity at higher sampling frequencies (10,000 Hz) and using post-processing software to separate a burst of sympathetic activity into individual action potentials [32,33]. Under baseline conditions, older adults demonstrate more action potentials per burst of sympathetic activity than young adults [34]. In response to strong sympathetic stimuli (maximal voluntary apnea), older individuals have a blunted rise in the firing probability of previously active action potentials (analogous to blunted rate-coding) coupled with attenuated recruitment of previously silent larger and faster conducting sympathetic action potential recruitment (analogous to blunted population-coding) relative to young participants [34]. Although sex differences were not investigated in their study, they are likely to exist, as work in young individuals has provided evidence that sex and the menstrual cycle impact sympathetic action potential recruitment patterns in a manner that is mediated, in part, by gonadal hormones [35].

In summary, there is a double hit to neurovascular physiology that potentiates the risk of hypertension in females around the age of menopause that is not observed in males. This double hit is characterized by both an increase in sympathetic activity and decreased β-adrenergic-mediated vasodilation that function, in tandem, to increase blood pressure around this age. Importantly, the role of sympathetic neural discharge patterns in the onset of these age-by-sex differences in blood pressure control is incompletely understood. Furthermore, despite clear sex differences in the underlying neurovascular physiology and established changes with aging, few studies have evaluated potential sex differences in neurovascular physiology following acute or chronic sleep disruption.

## 3. Sleep Disruption and Cardiovascular Risk: Sex Differences 

### 3.1. Epidemiological Data

Sleep is crucial to maintaining systemic homeostatic balance and is particularly impactful on cardiovascular regulation [1]. The prevalence of short sleep duration (i.e., total sleep time less than six or seven hours) was nearly 10% throughout the late 20th century and has increased during the early 21st century, when nearly one-third of the United States’ population habitually obtains short sleep [36,37]. Short sleep duration can originate from a self-imposed short sleep window or from a sleep pathology such as insomnia or OSA, both of which are characterized by short and/or fragmented sleep [38]. Nearly 20 years ago, Gottlieb et al. [39] provided the first epidemiological evidence connecting short sleep duration to the prevalence of hypertension. Objective short sleep duration in sleep disorders, such as insomnia [40,41] and OSA [42,43], are also associated with increased risk for developing conditions such as hypertension. 

Using epidemiological modeling and the Whitehall II study cohort, Cappuccio et al. [6] established a sex-specific relationship between chronic short sleep duration and hypertension. The relative risk of both prevalent and incident hypertension was 56% in females who habitually slept less than six hours, which increased to 94% in females who regularly slept less than five hours per night; importantly, this relationship was independent of age and not present in males. Data from the largest study sample to date (over 700,000 individuals) corroborate this notion, whereby Grandner et al. [7] found that the risk of hypertension associated with chronic short sleep duration is stronger in females throughout the lifespan as compared to age-matched males. One of the contributing mechanisms linking sleep disruption and the onset of hypertension, particularly with aging, is increased sympathetic nerve activity. Muscle sympathetic nerve activity is lower in premenopausal females compared to age-matched males, although this relationship is reversed in older age, as females exhibit a more robust increase in muscle sympathetic nerve activity per decade of life compared to males [19]. This is accompanied by the enhanced sympathetic support of blood pressure in postmenopausal females [30], suggesting that sympathetic dysregulation may contribute to hypertension risk more so in older females, coinciding with the peri/postmenopausal transition when sleep disorders become more prevalent [30].

### 3.2. Experimental Studies on Sleep Disruption

At present, only total sleep deprivation paradigms have utilized microneurography, the gold standard in vivo assessment of sympathetic nerve activity, and successfully discerned sex-specific alterations in the sympathetic response to experimental sleep perturbations. Early total sleep deprivation studies employed 24 h of sleep deprivation as a proof-of-concept model to delineate an acute increase in arterial blood pressure [30,44]. This acute rise in blood pressure was accompanied by a baroreflex-mediated decrease in muscle sympathetic nerve activity [44,45], although these study samples were exclusively [44] or predominantly [45] male. Carter et al. [5,22] employed a similar 24 h sleep deprivation model in young and older adults (1:1 female-to-male ratio) to elucidate potential sex differences in the regulation of blood pressure by sympathetic nerve activity. In both young and older adults, 24 h total sleep deprivation elicited an acute rise in blood pressure, consistent with prior studies [44,45]; however, the response in sympathetic nerve activity diverged between sexes. In young males, muscle sympathetic nerve activity was reduced following total sleep deprivation, in support of prior work by Kato et al. [45] and Ogawa et al. [44]. In contrast, young females exhibited a sympathetic predominance via baroreflex dysfunction (i.e., a rise in blood pressure not accompanied by reduced sympathetic nerve activity) with no differences observed in muscle sympathetic nerve activity between normal sleep and total sleep deprivation conditions. Older males, who are at risk for hypertension and sleep disorders, tended to exhibit a decrease in muscle sympathetic nerve activity despite a rise in blood pressure following total sleep deprivation [5]. In comparison, older females exhibited sympathoexcitation, suggesting that sympathetic dysregulation, perhaps secondary to baroreflex dysfunction, may be a driving factor in short sleep-mediated hypertension in postmenopausal females [5].

Other ecologically valid models of sleep disruption include partial sleep deprivation (e.g., sleep stage-specific disruption) and sleep restriction (e.g., curtailed opportunity to sleep). Sayk et al. [46] examined changes in next-morning sympathetic activity via microneurography following a single night of slow wave sleep deprivation induced by acoustic stimuli. In this study, slow wave sleep was targeted due to the known baroreflex resetting that occurs during this stage [47,48]. Surprisingly, the morning-after muscle sympathetic nerve activity and blood pressure regulation were not different between normal sleep and partial sleep deprivation conditions; unfortunately, the study was underpowered to discern sex differences. At present, Covassin et al. [4] remains the only sleep restriction study with adequate statistical power to delineate alternations in the sympathetic control of blood pressure using a randomized, crossover study design consisting of a nine-day, four-hour sleep per night restriction model. Sleep restriction elicited a sympathoexcitatory response indexed by increased plasma norepinephrine in both males and females. Moreover, this sympathoexcitation was accompanied by increased 24 h blood pressure selectively in females. To date, there have been studies that have characterized alterations in sympathetic nerve activity following partial sleep deprivation or sleep restriction.

Experimental models of total sleep deprivation, partial sleep deprivation, and sleep restriction have consistently provided mechanistic evidence linking disrupted sleep to cardiovascular risk vis a vis increasing blood pressure. More chronically, only Carter et al. [49] has measured muscle sympathetic nerve activity in a female-dominant group of people with insomnia compared to controls. Thus, it remains equivocal if sex differences in sympathetic dysregulation with insomnia are present. Similarly, heightened sympathetic nerve activity is well-established in patients with OSA, yet studies investigating sex differences are presently absent from the literature. This is at odds with existing experimental sleep curtailment paradigms showing potentiated risk in females, and future work is strongly warranted in this area.

## 4. Obstructive Sleep Apnea

Obstructive sleep apnea is identified by recurrent complete (apnea) or partial (hypopnea) airway collapses during sleep. The number of apneas and hypopneas are summed, indexed per hour of sleep, and used to identify and then characterize the severity of OSA. Specifically, 5.0–14.9 events per hour indicates mild OSA, whereas 15.0–29.9 events per hour is moderate OSA, and ≥30.0 events per hour is severe OSA [50]. Clinically, OSA is associated with a precipitous increase in the risk of stroke, coronary artery disease, and heart failure, with dysregulation of arterial blood pressure being a significant contributor to these comorbid cardiovascular diseases [51]. Indeed, approximately five-in-ten patients with OSA present to their initial diagnostic sleep study with hypertension [52]. Similarly, OSA predicts future diagnosis of hypertension independent of age, sex, body fat, and exercise habits [9]. In addition to elevated blood pressure during waking hours, patients with OSA also have nocturnal hypertension [53] and impaired nocturnal blood pressure dipping [54], both of which predict cardiovascular mortality [55,56]. Historically, OSA is male-dominant, although this is deleterious to women’s health as it promotes their underreporting to sleep clinics [55,56] and, ultimately, contributes to their underdiagnosis [57] and undertreatment [58]. Further contributing to this discrepancy are sex differences in the clinical phenotype and symptomology of females with OSA, which may inadvertently lead healthcare practitioners to pursue a differential diagnosis away from OSA [59,60,61]. Nevertheless, some evidence suggests females with OSA are more predisposed to develop hypertension than males [62], although this is inconsistently reported [63]. The notion that females with OSA could be at greater risk of hypertension than males with OSA is not without merit. Indeed, females with severe OSA have more frequent and more severe oxygen desaturations during the rapid eye movement stage of sleep as compared to males with OSA [23]. It is important to note that sympathetic nerve activity is greatest during rapid eye movement sleep [64]. Collectively, these studies indicate that the pathophysiological burden of OSA in females may be concentrated in the window of sleep when the sympathetic nervous system is most active, suggesting they may have greater sympathetic dysregulation and a greater risk of hypertension than males with OSA. While the contribution of hypertension to OSA-related cardiovascular disease is evident, there have been no studies to date that have investigated potential sex differences in the sympathetic regulation of blood pressure in patients with OSA. Here, we will provide a brief review of the sympathetic reflexes that contribute to hypertension in OSA, along with evidence supporting a sexual dimorphism in the sympathetic regulation of blood pressure in these patients. Then, we will discuss human studies that provide insight into potential mechanisms responsible for neurovascular dysfunction in patients with OSA while highlighting evidence of sex differences, when applicable.

### 4.1. Sympathetic Regulation of Blood Pressure in OSA

**Figure 2:**
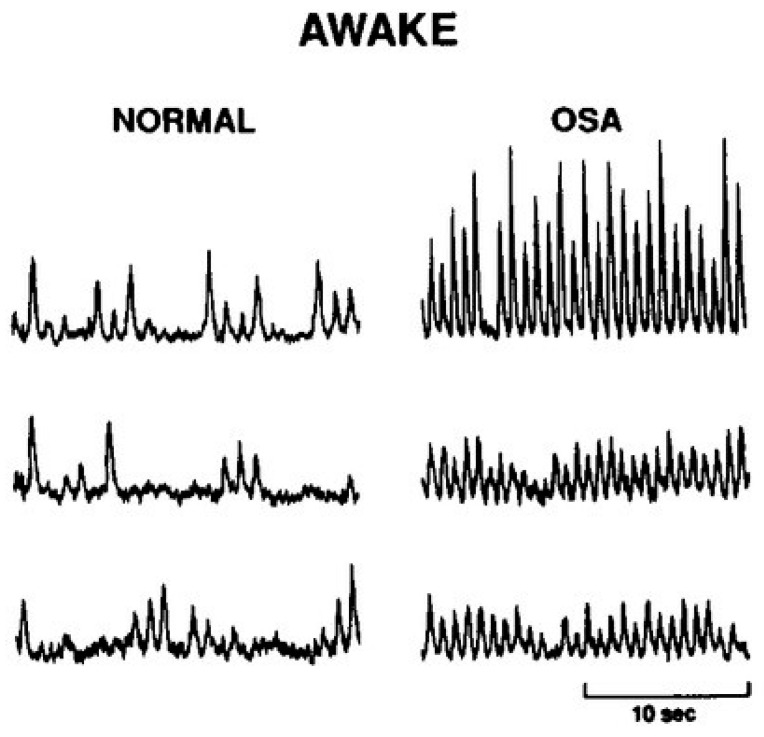
Sample neurograms recorded from the peroneal nerve under resting conditions from n = 3 patients with obstructive sleep apnea (OSA) as well as n = 3 individuals of similar age and body mass index who do not have OSA (Normal). These sample neurograms demonstrate a robust increase in sympathetic nerve firing rate and burst amplitude in patients with OSA. Reprinted from Somers et al. *J. Clin. Investig.* 1995 with permission [21].

**Figure 3:**
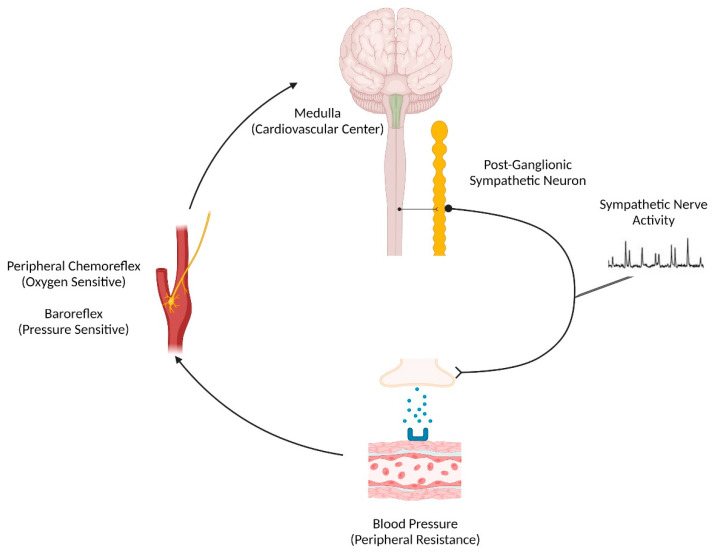
A schematic overview of the neural feedback mechanisms that regulate blood pressure. The sympathetic baroreflex operates as a negative feedback mechanism whereby increases in blood pressure (i.e., peripheral resistance) are sensed by stretch receptors located near the carotid bifurcation. These baroreceptors activate the caudal ventrolateral medulla, which functionally restricts the rostral ventrolateral medulla (RVLM), manifesting as attenuated sympathetic nerve activity [69]. Conversely, the sympathetic baroreflex increases sympathetic nerve activity, elevating peripheral resistance when arterial blood pressure declines. The peripheral chemoreflex is initiated when arterial PO_2_ falls as sensed by type 1 glomus cells in the carotid bodies, which, through the carotid sinus nerve, synapse in the RVLM to increase sympathetic nerve activity [70]. This increase in sympathetic nerve activity elevates arterial blood pressure via the binding of neurotransmitters to peripheral adrenergic receptors, functionally offsetting tissue-level hypoxic vasodilation, which preserves end-organ perfusion.

Several physiological systems regulate arterial blood pressure [65], although, in patients with OSA, the sympathetic nervous system is particularly influential. That is, patients with OSA have approximately 50% more sympathetic nerve bursts per minute relative to controls of similar age, sex, and body mass index (Figure 2) [24]. The baroreflex is our principal autonomic regulator of arterial blood pressure and functions via mechanical stretch receptors located in the carotid sinus (Figure 3). Activated during periods of blood pressure elevation, the baroreflex inhibits sympathetic outflow and, conversely, functions to elevate sympathetic nerve activity and peripheral resistance when blood pressure falls. Indeed, when these pressure receptors are unloaded pharmacologically (via intravenous nitroprusside infusion), patients with OSA have a blunted rise in sympathetic nerve activity indicative of a reduction in sympathetic baroreflex sensitivity [66]. Attenuated high-frequency heart rate variability [67], along with greater blood pressure variability [68], further indicate autonomic instability with OSA. While these data implicate baroreflex dysfunction as a contributor to the hypertensive phenotype of OSA, other studies suggest it is not the most significant autonomic determinant.

Oxygen-sensing chemoreceptors located in the carotid bifurcation and aortic arch principally respond to falls in PaO_2,_ reflexively increasing ventilation and sympathetic nerve activity. In humans, carotid chemoreceptors are the dominant oxygen sensors, as evidenced by the physiological responses to hypoxia being nearly abolished following surgical removal of the carotid bodies [71,72]. Seminal data from Narkiewicz et al. [73] illustrated that the sympathetic response to chemoreflex activation, measured through an end-expiratory apnea (to inhibit pulmonary afferent suppression of sympathetic nerve activity) following inspiratory hypoxia, is potentiated in patients with OSA as compared to controls. In a succeeding study, the investigators found that peripheral chemoreflex inhibition (inspiratory hyperoxia) reduced sympathetic activity by 17% and mean arterial pressure by 4 mmHg exclusively in patients with OSA, thus suggesting that tonic chemoreflex activation accounts for a portion of the increased basal sympathetic tone observed with OSA [24]. However, the physiological responses to central chemoreflex stimulation (inspiratory hypercapnia) and a cold pressor test (total sympathoexcitatory arch) are comparable between patients with OSA and controls [73]. Collectively, these data indicate the nocturnal intermittent hypoxemia induced by cyclical changes in airway patency precipitates the hypertensive phenotype typified in OSA via increasing sympathetic nerve activity. This notion has sparked interest in exploring the neurophysiological effects of acute intermittent hypoxia during waking hours in people with and without sleep-disordered breathing.

### 4.2. Intermittent Hypoxia Studies

For more than twenty years, intermittent hypoxia protocols have been used in healthy humans to mimic OSA [74] despite it being an imperfect replication of the pathophysiology [75]. Several studies have explored the cardiopulmonary responses to intermittent hypoxia as reviewed elsewhere [76]; however, far fewer have examined the effects of intermittent hypoxia on sympathetic nerve activity. It is important to note that the ventilatory and sympathetic responses to chemoreflex activation are differentially regulated [77], whereby changes in minute ventilation during hypoxia should not be interpreted as congruent with sympathetic nerve activity. Cutler et al. [78] conducted the earliest of these studies, finding that 20 min of intermittent hypoxia sensitizes the peripheral chemoreceptors for three hours following the final bout of hypoxia. A recent series of experiments from the Limberg group has furthered our understanding of the neurophysiological effects of intermittent hypoxia. For instance, Ott et al. [79] reported that the increase in sympathetic nerve activity following 30 min of intermittent hypoxia is a function of both greater intra-sympathetic burst action potential firing in addition to recruitment of more robust inter-burst action potentials. Two follow-up studies investigated the sex-specific effects of intermittent hypoxia on sympathetic activity. Principally, 30 min of intermittent hypoxia increases sympathetic nerve activity similarly in young, healthy males (Δ7.2 ± 1.8 bursts/100 heartbeats) and females (Δ5.5 ± 2.2 bursts/100 heartbeats), while blood pressure increases in males but decreases in females [80]. This novel finding suggests that young females may be resilient against the deleterious effects of intermittent hypoxia. Preclinical experiments support this notion, whereby the hypertensive effects of intermittent hypoxia were more pronounced in ovariectomized rodents relative to females that retained their ovaries [81]. A similar study illustrated that intermittent hypoxia sensitizes lung vagal C fibers to a greater extent in ovariectomized rats relative to those with intact ovaries and that this effect was offset with supplemental 17β-estradiol [82]. While the clinical implications of these findings are not immediately apparent, these data clearly indicate that intermittent hypoxia alters sympathetic discharge patterns differently between sexes and that female sex hormones appear to offset the neurophysiological effects of intermittent hypoxia. 

In contrast to these observations, human and preclinical data purport intermittent hypoxia may yield beneficial effects on vascular function. For instance, Iwamoto et al. [83] found that 50 min of intermittent hypoxia enhanced the endothelial function of the internal carotid artery by 33% in healthy young adults. A succeeding study reported the same intermittent hypoxia protocol partially offset the attenuation in brachial artery endothelial function associated with acute physical inactivity [84]. Fifteen days of intermittent hypoxia (~45 min per day) reduced systolic blood pressure by nearly 11 mmHg in patients with hypertension and OSA who were being treated with positive airway pressure therapy [85]. Data supporting a mechanism for this observation come from in vitro experiments that suggest intermittent hypoxia may blunt sympathetically induced transcriptional changes associated with the development of atherosclerosis [86]. Differentially, intermittent hypoxia was shown to induce autophagy via the AMPK/mTOR pathway, which may serve as a protective mechanism for endothelial cells in patients with OSA [87]. Another potential mechanism for the beneficial effects of intermittent hypoxia could be that the cyclical fluctuations in shear rate during intermittent hypoxia act on arterial endothelial cells to promote nitric oxide synthesis [83,88]. Indeed, elevating systemic nitric oxide levels improves blood pressure and autonomic function in patients with OSA [89].

Taken together, intermittent hypoxia is leveraged in research studies to mimic OSA, with increases in sympathetic nerve traffic being a frequently reported outcome. In recent years, it has become apparent that intermittent hypoxia influences the regulation of sympathetic nerve activity differently between males and females. Mild intermittent hypoxia also exerts beneficial effects on vascular health, which may be a function of peripheral vasodilation, as observed in skeletal muscle [90]. Although promising, caution should be used when interpreting these data, as inter-study differences in the hypoxic stimulus, maintenance of P_ET_CO_2_, the inclusion of females, and statistical power to detect sex differences make clinical extrapolation challenging. Indeed, greater “doses” of intermittent hypoxia (i.e., more severe hypoxemia, longer exposure) increase blood pressure while promoting oxidative stress and inflammation [91]. To this point, it is important to denote if the intermittent hypoxia protocol of interest is designed to mimic OSA or lower blood pressure, as this delineation likely explains discrepant findings between experimental studies and the etiology of OSA.

## 5. Future Directions

The body of evidence outlined above provides strong support for future investigations of potential sex differences in sympathetic cardiovascular regulation in patients with OSA. This series of studies should begin with exploring if peripheral chemoreflex and/or baroreflex sensitivity differ between males and females with OSA. It should be noted that recent data show young females have greater increases in sympathetic nerve activity during hypercapnia (central chemoreflex activation) relative to young males [92]. Thus, it may be that sex differences in neurovascular control in patients with OSA are a function of central versus peripheral chemoreflex sensitivity. Further, sympathetic action potential firing patterns may contribute to sex differences in sympathetic regulation in OSA. Therefore, it would be relevant to explore sex differences in neurovascular transduction to determine if a given burst of sympathetic nerve activity induces a greater vasoconstrictor response in females with OSA. It will be important to control for age during these studies, as β-adrenergic-mediated vasodilation is attenuated in older females [14], and the prevalence of OSA increases with age [93]. Collectively, it is evident that much more work on sex differences in patients with OSA is needed.

## 6. Summary

The deleterious effects of sleep disruption on cardiovascular risk, in the absence of overt sleep disorders, are more pronounced in females relative to males. Obstructive sleep apnea is among the most-studied chronic sleep disorders and is historically more prevalent in males than females. This has fueled the underrepresentation of females with OSA in studies, which subsequently limits our understanding of sex differences in the etiology and pathophysiology of OSA. As much of the cardiovascular risk associated with OSA is attributable to neurogenic hypertension, studies examining the neurovascular regulation of blood pressure in females with OSA are of critical importance. We hope this review serves as an impetus for the wider inclusion of females in sleep-related clinical research and the exploration of sex differences in the neurovascular responses to sleep disruption.
